# Phase Behavior and Role of Organic Additives for Self-Doped CsPbI_3_ Perovskite Semiconductor Thin Films

**DOI:** 10.3390/mi14081601

**Published:** 2023-08-14

**Authors:** Tamiru Kebede, Mulualem Abebe, Dhakshnamoorthy Mani, Jibin Keloth Paduvilan, Lishin Thottathi, Aparna Thankappan, Sabu Thomas, Sarfaraz Kamangar, Abdul Saddique Shaik, Irfan Anjum Badruddin, Fekadu Gochole Aga, Jung Yong Kim

**Affiliations:** 1 Faculty of Materials Science and Engineering, Jimma Institute of Technology, Jimma University, Jimma P.O. Box 378, Ethiopia; tamiruks@gmail.com (T.K.); mulualem.mekonnen@ju.edu.et (M.A.); dhakshnamoorthy.mani@ju.edu.et (D.M.); 2 Department of Physics, College of Natural and Computational Science, Bonga University, Bonga P.O. Box 334, Ethiopia; 3 School of Chemical Sciences, Mahatma Gandhi University, Kottayam 686560, India; jibinkp999@gmail.com; 4 Department of Physics and Mathematics, Università Cattolica del Sacro Cuore, Via della Garzetta, 48, 25133 Brescia, BS, Italy; lishint123@gmail.com; 5 Department of Physics, Baselius College, Kottayam 686001, India; aparnathankappan@baselius.ac.in; 6 School of Energy Materials, Mahatma Gandhi University, Kottayam 686560, India; sabuthomas@mgu.ac.in; 7 Mechanical Engineering Department, College of Engineering, King Khalid University, Abha 61421, Saudi Arabia; sarfaraz.kamangar@gmail.com (S.K.); abdul.siddique1976@gmail.com (A.S.S.); magami.irfan@gmail.com (I.A.B.); 8 Department of Materials Science and Engineering, Adama Science and Technology University, Adama P.O. Box 1888, Ethiopia; fekadu.gochole@astu.edu.et; 9 Center of Advanced Materials Science and Engineering, Adama Science and Technology University, Adama P.O. Box 1888, Ethiopia

**Keywords:** perovskite, organic additive, cesium lead iodide, self-doped, yellow δ-phase, black γ-phase, phase behavior

## Abstract

The phase change of all-inorganic cesium lead halide (CsPbI_3_) thin film from yellow δ-phase to black γ-/α-phase has been a topic of interest in the perovskite optoelectronics field. Here, the main focus is how to secure a black perovskite phase by avoiding a yellow one. In this work, we fabricated a self-doped CsPbI_3_ thin film by incorporating an excess cesium iodide (CsI) into the perovskite precursor solution. Then, we studied the effect of organic additive such as 1,8-diiodooctane (DIO), 1-chloronaphthalene (CN), and 1,8-octanedithiol (ODT) on the optical, structural, and morphological properties. Specifically, for elucidating the binary additive–solvent solution thermodynamics, we employed the Flory–Huggins theory based on the oligomer level of additives’ molar mass. Resultantly, we found that the miscibility of additive–solvent displaying an upper critical solution temperature (UCST) behavior is in the sequence CN:DMF > ODT:DMF > DIO:DMF, the trends of which could be similarly applied to DMSO. Finally, the self-doping strategy with additive engineering should help fabricate a black γ-phase perovskite although the mixed phases of δ-CsPbI_3_, γ-CsPbI_3_, and Cs_4_PbI_6_ were observed under ambient conditions. However, the results may provide insight for the stability of metastable γ-phase CsPbI_3_ at room temperature.

## 1. Introduction

Metal halide perovskites (MHPs) have the general formula of ABX_3_, where A is methyl ammonium (MA) CH_3_NH_3_^+^, formamidinium (FA) CH(NH_2_)_2_^+^, methylhydrazinium CH_3_(NH_2_)_2_^+^, aziridinium (CH_2_)_2_NH_2_^+^, cesium (Cs), or rubidium(Rb); B is lead (Pb), tin (Sn) or manganese (Mn); and X is halide (Cl, Br, I) or its mixture [1,2,3,4]. MHPs can serve as a semiconducting active layer for photovoltaic (PV) cells, photodetectors (PD), light-emitting diodes (LEDs), field-effect transistors (FETs), and sensors [5,6]. Due to the thermal instability of organic cations, all-inorganic cesium lead halide (CsPbX_3_, X = Cl, Br, I) becomes an alternative material by incorporating the inorganic cesium instead of MA or FA [7,8,9,10,11]. CsPbX_3_ is known to have long charge carrier diffusion length, strong light absorption, defect tolerance, thermal tolerance, narrow spectral bandwidth, tunable direct bandgap, high photoluminescence quantum yields (PLQY), and solution/melt processability [12,13,14,15,16,17,18,19,20,21,22,23,24]. The power conversion efficiency (PCE) of all-inorganic CsPbX_3_ perovskite solar cells (PeSC) has reached ~21.15% [25] whereas the best PCE of FAPbI_3_ solar cell is ~26% in 2023 [26].

Among cesium lead halides, the cubic phase α-CsPbI_3_ has the smallest tolerance factor $t=\left(r_{A}+r_{X}\right)/\left\{\sqrt{2}\cdot \left(r_{B}+r_{X}\right)\right\}$ = 0.805, where $r_{A}$, $r_{B}$, and $r_{X}$ are the radius of cation A, cation B, and anion X, respectively [27,28]. This *t* value slightly falls out from the structural stability condition of 0.813 ≤ *t* ≤ 1.107, indicating that α-CsPbI_3_ may undergo a rapid phase transformation below 320 °C [29]. Hence, α-CsPbI_3_ (black cubic) is structurally unstable and converted into β-CsPbI_3_ (black tetragonal), γ-CsPbI_3_, (black orthorhombic), and δ-CsPbI_3_ (yellow orthorhombic) at room temperature [30,31,32]. Specifically, δ-CsPbI_3_ is non-perovskite but thermodynamically most stable at room temperature, suggesting that this phase should be avoided for perovskite optoelectronics. However, because of its energy bandgap (*E*_g_) of 1.73 eV [11] affording significant photon harvesting, CsPbI_3_ has received more attention than the other cesium-led halides (CsPbCl_3_ with *E*_g_ = 3.03 eV and CsPbBr_3_ *E*_g_ = 2.23 eV) for PeSC applications [33,34,35,36]. In this process, the researchers tried to overcome the intrinsic phase instability of CsPbI_3_ through additive engineering, quantum dots, dimension engineering, composition engineering, metal ion doping, solvent engineering, surface/defects passivation, and interfacial engineering [37,38,39,40,41,42,43,44].

In 2015, Snaith and coworkers demonstrated the working all-inorganic CsPbI_3_ solar cell for the first time, in which hydroiodic acid (HI) was identified to stabilize γ-CsPbI_3_ at a relatively lower temperature, ~100 °C [45]. Marronnier et al. observed the temperature-dependent phase transformation from orthorhombic to cubic (δ→α) upon heating but cubic–tetragonal–orthorhombic (α→β→γ) upon undercooling, indicating that CsPbI_3_ could temporally retain its black γ-phase at room temperature [46]. Zhang et al. improved the crystal structure stability of γ-CsPbI_3_ through interface engineering by depositing γ-CsPbI_3_ on top of iodine-doped reduced graphene oxide [47]. Wang et al. enhanced the γ-phase CsPbI_3_ stability and minimized trap density by controlling crystallization dynamics using chlorine doping [48]. Liu and coworkers demonstrated that the γ-phase could be stabilized by reducing defect densities acting as both recombination center and ion migration space, for which they employed an acyloin ligand (1,2-di(thiophen-2-yl)ethane-1,2-dione (DED)) as a phase stabilizer and defect passivator [25]. Huang et al. recognized that the intrinsic instability of the γ-phase originates from the small ion radius of cesium. Hence, to solve this problem, they incorporated small amounts of poly(alkyl amine hydrochloride) (PAACl) additive to the perovskite precursor solution and improved the stability of γ-phase CsPbI_3_ [49]. Vaynzof and coworkers demonstrated the fabrication of a relatively stable γ-CsPbI_3_ thin film through co-evaporation of CsI and PbI_2_ with a small amount of phenylethylammonium iodide (PEAI), affording a preferable crystal orientation (columnar domains) with reduced defect densities [50]. Recently, Zhou and coworkers identified the excess CsI itself (i.e., more than 1 = CsI/PbI_2_) preferred a formation of black γ-phase to yellow δ-phase, which is interesting in that it used a self-component rather than external one [51].

Additive engineering has been frequently employed for enhancing the performances of both polymer solar cells (PSCs) and PeSCs [38,52,53]. In the case of PSCs, the phase-separation scale should be controlled within the exciton diffusion length (~10–47 nm depending on fullerene or non-fullerene acceptor and conjugated polymer) [54,55,56]. On the other hand, in the PeSCs, it is important to control the nucleation and crystal growth of perovskite from the colloidal dispersion via intermediate phase engineering (IPE) [57,58], which is used for obtaining a high-quality perovskite layer (ideally a single crystal but practically a polycrystal with minimized defects). Specifically, 1,8-diiodooctane (DIO), 1-chloronaphthalene (CN), and 1,8-octanedithiol (ODT) have been commonly used for organic electronics [59,60,61,62,63].

In 2007, Heeger and coworkers demonstrated that the addition of a few volume percent of alkanedithiols including ODT has contributed to the enhancement of PCE from 2.8% to 5.5% through improving the bulk heterojunction morphology of PSCs [64,65]. Then in the next year, the same group identified that DIO was the best among 1,8-di(R)octanes (R: SH, Cl, Br, I, and CO_2_CH_3_) and suggested two criteria: (a) selective solubility of the electron acceptor and (b) high boiling point of additive [65]. Then, to date, this additive strategy has been continuously applied for non-fullerene-acceptor (NFA)-based PSCs as well as all-polymer solar cells (all-PSCs) [59,66]. However, in the field of PeSCs, the conventional solvent additives such as DIO, CN, and ODT are relatively less studied for all-inorganic PeSCs although there are some for the hybrid PeSCs [67,68,69,70,71]. For example, in 2014, Jen and coworkers demonstrated that the bidentate halogenated additive, DIO can enhance the crystallization of MAPbCl_3_ [67]. In 2015, Chen and coworkers proved that the CN additive is beneficial to regulating the crystallization of MAPbI_3−x_Cl_x_ [68]. In 2018, Peng et al. observed that the DIO additive also could enhance the crystallinity of MAPbI_3−x_Cl_x_ [69]. In the same year, Tsai et al. proved that DIO is useful for the crystallinity, coverage, and uniformity of the MAPbI_3_ thin film for PeSCs [70]. Then, recently, Ghorai et al. reported the ligand-mediated revival of degraded α-phase CsPbI_3_ nanocrystals by using 1-dodecanethiol (DSH), in which a heavily distorted α-CsPbI_3_ could be converted to the cubic CsPbI_3_ phase via the trigonal Cs_4_PbI_6_ through the etching with the surface ligand/passivator, DSH [71].

In this study, we studied an all-inorganic CsPbI_3_ perovskite thin film doped with the excess cesium iodide with molar ration, CsI/PbI_2_ = 2, which was inspired by Zhou et al.’s interesting results with CsI/PbI_2_ = 1, 1.05, 1.5, and 4 in a nitrogen-filled glove box [51]. However, in our case, we carried out all the experiments in an ambient condition, indicating that the results may suggest air processibility and stability for the CsPbI_3_ thin film. However, note that, compared to N_2_ environment in a glove box, if we process the perovskite thin film in air, the humidity (H_2_O molecules) may affect the crystallization process of perovskite intermediates, which was explained by Lin and coworkers in detail [72]. Then, we examined the organic additive (DIO, CN, and ODT) effects on the structural, optical, and morphological properties of the self-doped CsPbI_3_ thin film for the first time. Hence, through this work, the dual effects, self-dopant and external additives, can be elucidated in air. Furthermore, we report the phase behavior of a binary additive–solvent system for the first time based on the Flory–Huggins theory, presenting the role of additive in a typical solvent (DMF and DMSO) medium used for perovskite electronics.

## 2. Materials and Methods

### 2.1. Materials

The materials used for the experimental works are lead iodide (PbI_2_, 99.99%%, Sigma-Aldrich, Darmstadt, Germany), cesium iodide (CsI, 99.99%, Sigma-Aldrich, Darmstadt, Germany), DMF (99.5%, AR chemicals, Delhi, India), DMSO (99%, AR chemicals, Delhi, India), chlorobenzene (≥99.5%, AR chemicals, Delhi, India), 1,8-diiodooctane (DIO, 98%, TCI chemicals, Tokyo, Japan), 1-chloronephtaline (CN, 99%, TCI chemicals, Tokyo, Japan), and 1,8-octanediithiol (ODT, 95%, TCI chemicals, Tokyo, Japan), which were used as received without further purification.

### 2.2. Methods

The perovskite precursors (0.8 mmol CsI and 0.4 mmol PbI_2_ without/with organic additives) were dissolved in the solvent mixtures of 600 μL DMF and 400 μL DMSO and stirred overnight at room temperature. Here, the additive was DIO, CN, or ODT, which was 2% of the DMF/DMSO-mixed solvents by volume. Then, the perovskite precursor solution was filtered using a polytetrafluoroethylene (PTFE) syringe filter with 0.22 μm pore size. Then, 70 μL of colloidal perovskite precursor dispersion was dispensed on the top of ITO glass substrate. Here, the spin coating was processed with 1000 rpm for 10 s and then 4000 rpm for 40 s. During spinning (after ~20 s), 200 μL CB antisolvent was dispensed on top of the wet perovskite precursor film. Then, the thin film samples were gently annealed on a hotplate at 120 °C for 10 min and cooled down to room temperature for further characterization. Note that Zhou et al. [51] annealed their thin film at 320 °C (i.e., a phase transition temperature for black α-phase CsPbI_3_ with cubic structure) for 3 s in a N_2_-filled glove box. However, we processed our thin film in air without transferring it to a glove box because we have interest in the air stability of all-inorganic CsPbI_3_ samples.

### 2.3. Characterization

The ultraviolet-visible (UV-vis) absorption data were obtained using UV-vis spectroscopy (SHIMADZU UV-2600, Kyoto, Japan). The photoluminescence (PL) emission spectra of the self-doped CsPbI_3_ thin films were acquired using a spectrophotometer (SHIMADZU RF-6000, Kyoto, Japan) at an excitation wavelength of 420 nm. The PL decay curves were recorded by using time-correlated single-photon counting (TCSPC) (model: Fluorolog 3 TCSPC, Horiba, Houston, TX, USA). The transmission electron microscopy (TEM) images were obtained by using a high-resolution TEM (HR-TEM, Model: JEOL, JEM-2100, Peabody, MA, USA) with an operating voltage of 200 kV. The structural properties of the self-doped CsPbI_3_ thin films were investigated by using an X-ray diffraction (XRD) analyzer (model: the Rigaku mini flex-300/600 diffractometer, Tokyo, Japan). The microstructural morphologies of the thin films were characterized by using field emission scanning electron microscopy (FE-SEM, MAIA 3XMH TESCAN, Kohoutovice, Czech Republic). The atomic force microscopy (AMF) tapping-mode images were obtained by using the Park NX10 AFM (Park Systems, Suwon, Republic of Korea). Fourier transform infrared spectroscopy (FT-IR) analysis was performed in transmittance mode by using the PerkinElmer Spectrum Two FT-IR Spectrometer (Waltham, MA, USA). Here, the attenuated total reflection (ATR) was employed to record the FT-IR spectra of the self-doped CsPbI_3_ thin films without/with organic additives in the range 4000–400 cm^−1^ with a resolution of 4 cm^−1^ [73].

### 2.4. Computational Methods

The electronic band structures of the unit cells (δ-CsPbI_3_ and γ-CsPbI_3_) were calculated using Cambridge Serial Total Energy Package software (CASTEP, Materials Studio 2017, Vélizy-Villacoublay, France). The Perdew–Burke–Ernzerhof (PBE) parametrization of the generalized gradient approximation (GGA) was used to portray the exchange correlation functional. The unit cell in the Brillouin zone was employed to estimate the electronic band structures. For geometry optimization, the energy, maximum force, maximum displacement, and maximum stress were 5 × 10^−5^ eV/atom, 0.01 eV/Å, 5 × 10^−4^ Å, and 0.02 GPa, respectively.

## 3. Results and Discussion

**Figure 1:**
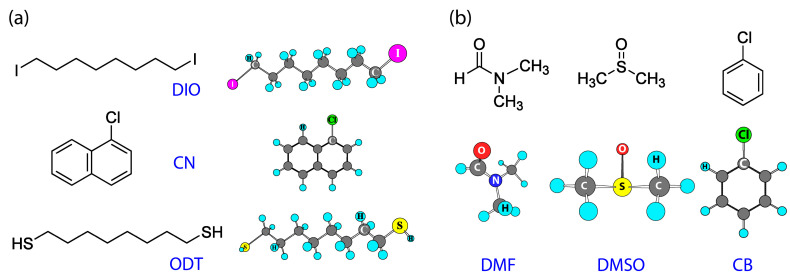
Chemical structures of organic additives and solvents. (**a**) Organic additives: DIO, CN, and OTD. (**b**) Solvents: DMF, DMSO, and CB.

Figure 1 shows the chemical structures of (a) additives (DIO, CN, and ODT) and (b) solvents (DMF, DMSO, and CB). Here, CB was used as an antisolvent during the solvent engineering process. Table 1 and Table 2 summarize the properties of additives and solvents, respectively. Specifically, DMF and DMSO have Gutmann’s donor number (*D_N_*) of 26.6 kcal/mol and 29.8 kcal/mol whereas CB has *D_N_* = 3.3 kcal/mol, indicating that DMF and DMSO can have strong coordination bonding with haloplumbate containing Lewis acid Pb^2+^ but CB cannot [74]. Therefore, the less polar CB molecule could act as an antisolvent, which has a weaker basicity as well as a smaller solubility parameter (*δ*) of 9.5 (cal/cm^3^)^1/2^ than the other two solvents (DMF and DMSO) [73,75].

In this study, for preparing a perovskite precursor solution, we used mixed solvents composed of DMF:DMSO = 3:2 volume ratio according to the literature report [79]. Then, we added DIO, CN, or ODT as a solvent additive into the perovskite precursor solution, resulting in a change in solvent quality, good or poor. Hence, we investigated the phase behavior of binary additive–solvent systems. Note that although we used a mixed DMF:DMSO solvent system, we should decouple it for understanding phase behavior theoretically.

The Flory–Huggins theory can describe polymer solution thermodynamics [80,81,82]. In this study, the processing solvent additives can be treated as an oligomer. Note that oligomer has a molecular weight whose degree of polymerization ≤10. Compared to the solvents such as DMF and DMSO, the molecular size of the organic additives (DIO, CN, and ODT) falls into the oligomer level (see Table 1 and Table 2). Hence, for the binary additive-solvent mixture modeled as an oligomer–solvent system, the Gibbs free energy of mixing could be expressed as Equation (1) according to Flory–Huggins theory [80],

(1) $$\frac{\Delta G_{mix}}{RT}=\frac{\phi_{1}}{r_{1}}\ln\phi_{1}+\frac{\phi_{2}}{r_{2}}\ln\phi_{2}+\chi_{12}\phi_{1}\phi_{2}$$

where $\phi_{1}$, $\phi_{2}$, $r_{1}$, and $r_{2}$ are the volume fraction and relative molar volumes of components 1 (solvent) and 2 (additive), respectively. In addition, *R* (=1.987 cal/(K·mol)) and *T* (K) are the gas constant and temperature, respectively. Importantly, the $\chi_{12}$ interaction parameter could be defined as $\chi_{12}={\hat{V}}_{1}/RT{\left(\delta_{1}-\delta_{2}\right)}^{2}$ [80,83], where ${\hat{V}}_{1}$ is the molar volume of solvent whereas $\delta_{1}$ and $\delta_{2}$ are the solubility parameter of component 1 and 2, respectively. Table 3 shows the $\chi_{12}$ and $r_{2}$ for each system when $r_{1}=1$ for a solvent (DMF or DMSO). Then, the binodal curve can be calculated based on the below two equilibrium equations.

(2) $$\Delta \mu_{1}^{\alpha }=\Delta \mu_{1}^{\beta }$$

(3) $$\Delta \mu_{2}^{\alpha }=\Delta \mu_{2}^{\beta }$$

where $\Delta \mu_{i}=\partial \Delta G_{mix}/\partial n_{i}$ is the chemical potential of component, i (=1, 2), and α and β are oligomer-lean phase and oligomer-rich phase, respectively [80,81,82,83].

**Figure 2:**
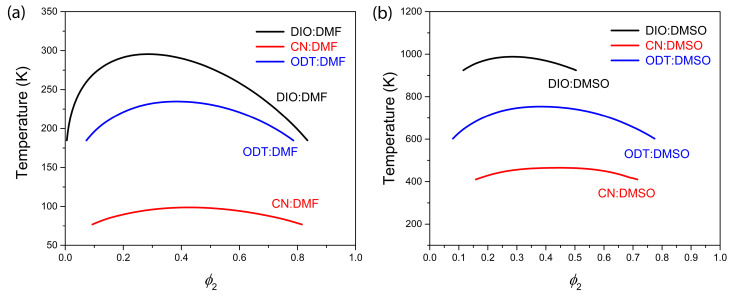
Phase diagrams of binary additive–solvent systems: (**a**) additive–DMF system and (**b**) additive–DMSO system. Here, the additive could be DIO, CN, or ODT, which has a molar volume with oligomer level compared to typical solvents such as DMF and DMSO.

Figure 2 shows the temperature–composition phase diagrams (i.e., the binodal curves) of (a) additive:DMF and (b) additive:DMSO systems, which were constructed by solving Equations (2) and (3) simultaneously. First of all, the original Flory–Huggins theory can capture a big essential picture without losing the physical meaning [80], indicating that we should understand the predicted phase behavior qualitatively, not quantitatively. Second, as indicated in Table 3, the phase behavior is largely governed by $\chi_{12}\;\mathrm{and}\;r_{2}$. Third, small $\chi_{12}$ denotes better miscibility between additive and solvent, indicating that the additive–DMF system is better miscible than the additive–DMSO system (see Table 3). In Figure 2, the additive–solvent miscibility has a similar sequence for both solvents, (a) CN:DMF > ODT:DMF > DIO:DMF for the additive–DMF solution, and (b) CN:DMSO > ODT:DMSO > DIO:DMSO for the additive–DMSO solution. However, the additive–DMF solution shows the immiscibility region below room temperature (<300 K) whereas the additive–DMSO solution displays it at a higher temperature (<1000 K, theoretically), indicating that the additive–DMF system has a better miscibility than the additive–DMSO mixture. Hence, in the DMF:DMSO = 3:2 mixture, if we employ an organic additive, the solvent quality is going to be poor specifically because additive is not much miscible with DMSO. Hence, we can expect that versatile iodide plumbate (${\mathrm{PbI}}_{n}^{2-n}$ with *n* = 2–6) are going to be more aggregated (i.e., self-interactions are increased) if the additive is present in the perovskite precursor solution. Here, it is noteworthy that (1) the perovskite precursor solution is a colloidal dispersion, and (2) DMF and the DMF–DMSO mixture are retrograde solvent systems [84,85]. Hence, the addition of organic additive has a similar effect on the rise in temperature in the sense that the self-interactions among haloplumbate are enhanced, which may affect the perovskite crystallization process, resulting in a different morphology of the final perovskite thin films. See Figure S1 and Table S1 in Supplementary Materials for the Flory–Huggins interaction parameter at 298 K (=25 °C).

**Figure 3:**
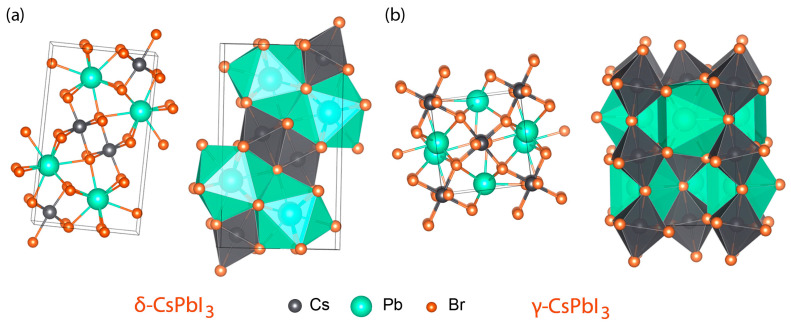
Crystal structures of (**a**) yellow δ-phase CsPbI_3_ and (**b**) black γ-phase CsPbI_3_.

At room temperature, the thermodynamically stable phase for CsPbI_3_ is yellow δ-phase [11,31,32]. However, when the temperature is increased to 587 K, it can undergo a phase transition into the black α-phase [31,32,33,34]. Then when α-phase is cooled down, it can transform to β-phase at 554 K and to γ-phase at 457 K. Hence, at room temperature, CsPbI_3_ could stay in yellow δ-phase (Figure 3a) or black γ-phase (Figure 3b), which are both orthorhombic. In this work, to escape the yellow δ-phase (i.e., non-perovskite), we added excess CsI into the perovskite precursor solution, which we call ‘self-doping’ because the excess CsI can serve as an interstitial dopant or stay at the surface of perovskite crystals. However, when we add excess CsI, it is known that Cs_4_PbI_6_ could be formed together with γ-phase CsPbI_3_ [86] (see Figure S2 for the trigonal phase of Cs_4_PbI_6_). For clarification, at this moment, it is noteworthy that in the field of conjugated polymers, ‘self-doping’ indicates that charge injected into the π–electron system of a conjugated polyelectrolyte with the potential counterions is compensated by cation (or anion) migration, leaving behind the negatively (or positively) charged counterions [87,88]. However, here we use ‘self-doping’ for the case of the perovskite doped with its own component (e.g., interstitial doping and/or surface passivation).

**Figure 4:**
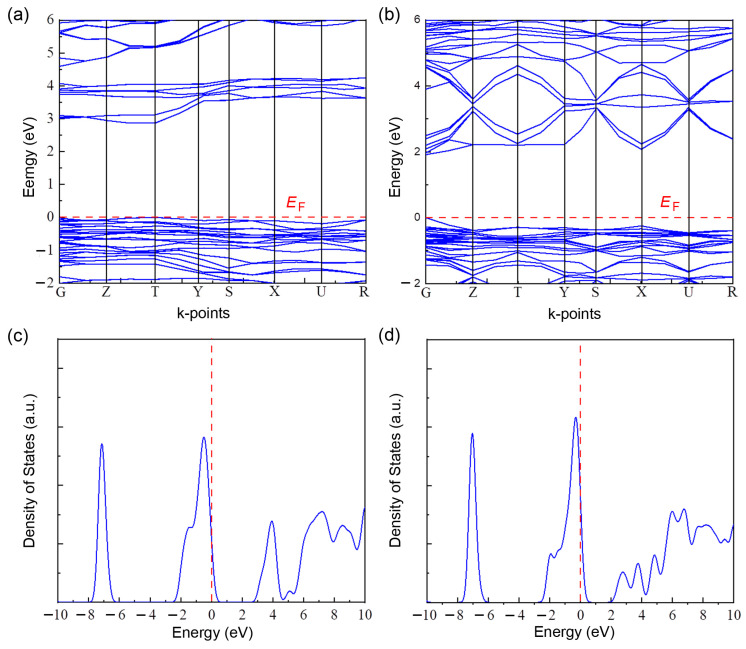
Electronic band structures: (**a**) yellow δ-phase CsPbI_3_ with the energy bandgap, *E_g_* = 2.87 eV and (**b**) black γ-phase CsPbI_3_ with *E_g_* = 1.90 eV. Density of states: (**c**) yellow δ-phase CsPbI_3_ and (**d**) black γ-phase CsPbI_3_. Here, *E_F_* stands for Fermi energy. Here, the red dot line is y = 0 for (**a**) and (**b**) and x = 0 for (**c**) and (**d**), respectively.

Figure 4a,b show the electronic band structure for (Figure 4a) yellow δ-phase and (Figure 4b) black γ-phase CsPbI_3_, which was calculated based on the unit cell structure shown in Figure 3. Figure 4c,d display the density of states for structure for δ-phase and γ-phase CsPbI_3_, respectively. Here, the estimated energy bandgap is 2.87 eV for δ-phase and 1.90 eV for γ-phase, respectively. The results are slightly larger than the experimental values explained below.

**Figure 5:**
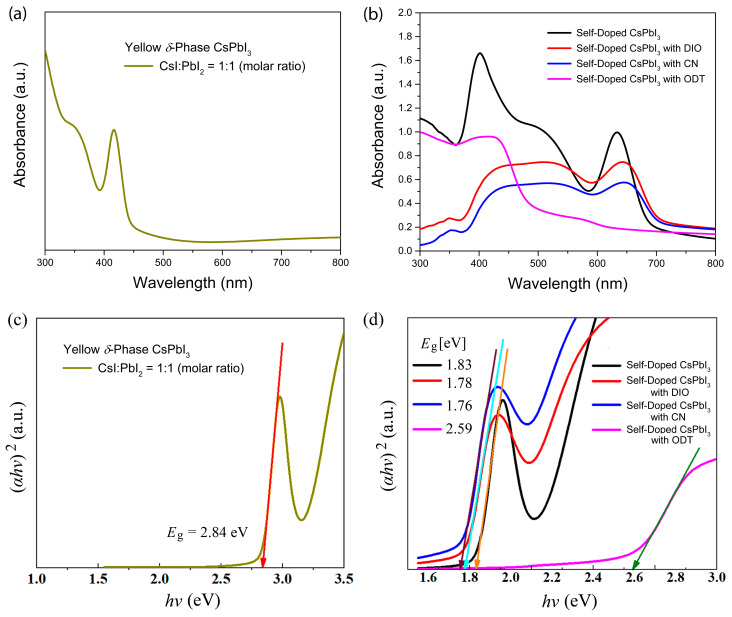
UV-vis absorption spectra of (**a**) yellow δ-phase CsPbI_3_ and (**b**) black γ-phase CsPbI_3_ and its derivative (e.g., Cs_4_PbI_6_ or low dimensional structure). Tauc plot for determining bandgap of (**c**) yellow δ-phase CsPbI_3_ and (**d**) black γ-phase CsPbI_3_ and its derivative. Here, the arrows indicate a tangential line to determine the optical bandgap.

Figure 5a,b show UV-vis absorption spectra for (a) the yellow δ-phase CsPbI_3_ and (b) the self-doped CsPbI_3_ without/with three organic additives (DIO, CN, and ODT). As shown in Figure 5a, when CsI:PbI_2_ = 1:1 under ambient conditions, the resulting structure is a yellow δ-phase orthorhombic crystal (recall Figure 3a). On the other hand, Figure 5c,d display the determination of bandgap based on Tauc plot, ${\left(\alpha h\nu \right)}^{2}$ vs. hν, where α is absorption coefficient, h is Plank’s constant, and ν is the frequency of incident photon [89]. Resultantly, the yellow δ-phase CsPbI_3_ exhibits an optical bandgap ($E_{g}$) of 2.84 eV at the wavelength (λ, i.e., absorption edge) of 437 nm whereas the self-doped γ-phase CsPbI_3_ displays $E_{g}$ = 1.83 eV. However, when the organic additive (DIO, CN, and ODT) was incorporated into the perovskite precursor solution, the resulting film shows different energy bandgaps like $E_{g}$ = 1.78 eV at λ = 697 nm for DIO, $E_{g}$ = 1.76 eV at λ = 705 nm for CN, and $E_{g}$ = 2.59 eV at λ = 479 eV, confirming that the presence of additive affected the crystallization process of perovskite (γ-phase) and non-perovskite (δ-phase). Here, it is notable that when the organic additive (DIO or CN) was incorporated into the film, $E_{g}$ was reduced from 1.83 eV to 1.78 eV (DIO) or 1.76 eV (CN). This reduction suggests that the internal crystal structures of thin films were better organized when the additive was present in the perovskite precursor solution. On the contrary, when the organic additive CN was employed for the perovskite film process, $E_{g}$ was widened from 1.83 eV to 2.59 eV (but still smaller than $E_{g}$ = 2.84 eV of yellow δ-phase), indicating that the contribution of γ-phase CsPbI_3_ might be minimized in this sample. Here, we guess that if ODT is ionized (R–SH→R–S^+^ + H^+^) in the perovskite precursor solution, the octacarbon chainlike cation (-S^+^) may partly increase the *d*-spacing of perovskite layers just like low-dimensional perovskite [90,91,92,93] and/or the accompanying polarity change in a colloidal dispersion medium may induce the formation of more δ-CsPbI_2_ and Cs_4_PbI_6_ with a larger bandgap under ambient conditions.

**Figure 6:**
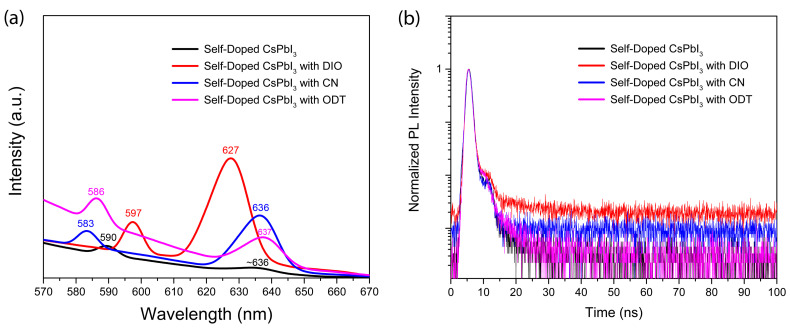
Self-doped CsPbI_3_ without/with organic additive: (**a**) PL emission spectra and (**b**) time-resolved PL decay spectra.

Figure 6a shows the PL spectra of self-doped CsPbI_3_ without/with organic additives. Interestingly, the PL spectra exhibit two different peaks at ~636 nm and 590 nm for the self-doped CsPbI_3_ without additive (the black solid line in Figure 6a). However, when DIO, CN, and ODT were added for the self-doped CsPbI_3_, the PL peak positions were shifted to 627 nm/597 nm (DIO), 636 nm/583 nm (CN), and 637 nm/586 nm (ODT), respectively. Here, it is interesting that the PL peak position of ODT-added sample is comparable to those of others, which is different from the results of UV-vis spectra in Figure 5b. One possibility is that in Figure 5b, there is a small bump around ~600 nm, which might be partially linked to the above PL emission. Here, it is noteworthy that the environmental condition was air (not a controlled glove box), making the samples exhibit diverse characteristics (i.e., the degree of internal phase transformation might be different). Figure 6b displays the time-resolved PL (TRPL) decay curve of self-doped CsPbI_3_ without/with organic additives. First of all, the decay curve shows two steps, i.e., a normal decay and additional small bump, which makes the regular model (single, double, triple exponential fitting) not suitable for describing the PL decay data. Hence, according to the literature report [90], we estimate the PL lifetime at the 63% decay point in Figure 6b. Resultantly, the PL lifetime is ~1.1 ns (self-doped CsPbI_3_, DIO, and CN) and ~0.9 ns (for self-doped CsPbI_3_ with ODT). However, as shown in Figure 6b, there are PL decay tail curves, indicating that DIO > CN > ODT ≈ ‘without additive’. Therefore, it seems that the additive engineering contributed to the slight enhancement in PL lifetime.

**Figure 7:**
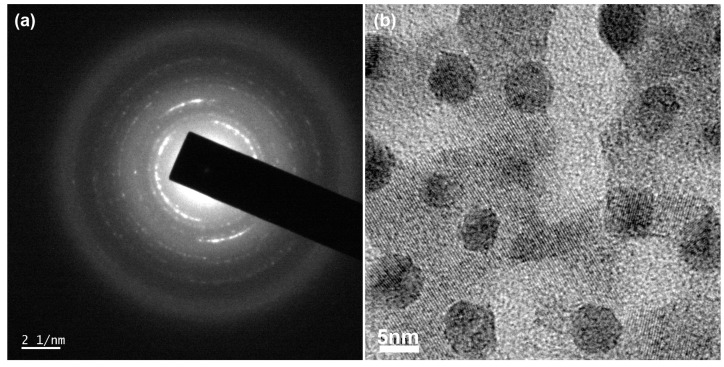
(**a**) Selected area diffraction pattern and (**b**) high-resolution TEM image of the self-doped CsPbI_3_ sample.

Importantly, for understanding both the two PL peaks and the two-step PL decay, we examined a high-resolution TEM (HR-TEM) image for the self-doped CsPbI_3_. As shown in Figure 7, the selected area diffraction pattern (Figure 7a) as well as the high-resolution TEM image (Figure 7b) could be identified, displaying the nanocrystals embedded in the crystalline matrix. Hence, we assume that these nanostructures might be related with the two PL peaks as well as the two-step TRPL decay curves. As an example, we selectively checked another sample, the self-doped CsPbI_3_ with the additive ODT; the result can be found in Figure S3, displaying a similar nanostructural image, i.e., the nanocrystal domains embedded in the crystalline matrix.

**Figure 8:**
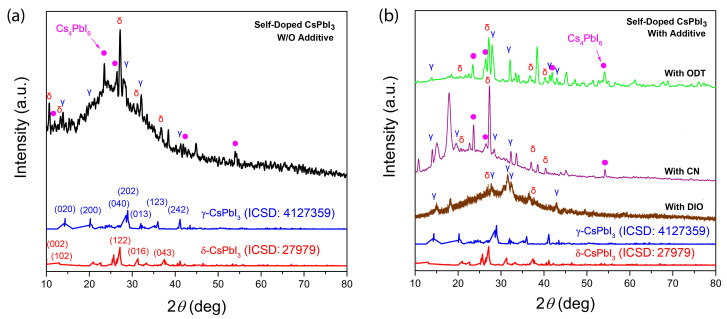
XRD patterns for (**a**) self-doped CsPbI_3_ without organic additive and (**b**) self-doped CsPbI_3_ with organic additives such as DIO (brown), CN (violet), and ODT (green). Note that the blue solid line is for γ-CsPbI_3_ from the Inorganic Crystal Structure Database (ICSD-4127359) whereas the red solid line is for δ-CsPbI_3_ from ICSD-27979. The pink filled circles indicate the XRD peaks from the trigonal Cs_4_PbI_6_ phase [95].

Figure 8a shows the XRD pattern of self-doped CsPbI_3_ without additive whereas Figure 8b displays the XRD patterns of self-doped CsPbI_3_ with the organic additives, DIO, CN, and ODT, which may highlight the results of air-processed cesium lead iodide samples. First of all, when CsPbI_3_ was prepared with excess CsI, we observed γ-phase CsPbI_3_ with an orthorhombic structure. However, at room temperature, the most stable structure is unfortunately δ-phase CsPbI_3_. Specifically, when γ-phase CsPbI_3_ is exposed to a humid condition, it is known that it transforms into the stable δ-phase CsPbI_3_ (non-perovskite, as shown in Figure 3a) [94]. Furthermore, the excess CsI compounds afford the formation of the trigonal Cs_4_PbI_6_ phase. Hence, as shown in Figure 8a, the self-doped CsPbI_3_ shows the XRD patterns mainly originating from three compounds such as γ-/δ-phase orthorhombic CsPbI_3_ and trigonal Cs_4_PbI_6_ [95]. Note that in this study, the reaction condition was 2CsI + PbI_2_, which could be a source for the reactions of CsI + PbI_2_ and 4CsI + PbI_2_, producing γ-/δ-CsPbI_3_ and Cs_4_PbI_6_ and others. Interestingly, Cs_4_PbI_6_ is known to be used for synthesizing CsPbI_3_ compounds [86]. Hence, as shown in Figure 8a, the self-doped CsPbI_3_ sample’s black γ-phase exhibits XRD peaks at 2*θ* = 13.9°, 19.9°, 28.1°, 32.1°, and 41.3°, corresponding to the orthorhombic crystallographic planes, (020), (200), (040), (013), and (242), respectively. On the other hand, its yellow δ-phase displays the XRD peaks at 2*θ* = 10.6°, 13.2°, 27.2°, 31.2°, and 36.8°, corresponding to the crystallographic planes, (002), (102), (122), (016), and (043), respectively. Note that for this peak assignment, referring to the Inorganic Crystal Structure Database (ICSD), 4,127,359 and 27,979 were used for γ-phase and δ-phase, respectively. In the case of Cs_4_PbI_6_, the XRD peaks are observed at 2*θ* = 12.0°, 23.4°, 26.4°, 42.0°, and 53.9°, corresponding to the trigonal crystallographic planes, (012), (300), (131), (060), and (354), respectively [91]. Figure 8b shows the XRD patterns for the self-doped CsPbI_3_ with versatile organic additives, DIO (brown), CN (violet), and ODT (green solid line). As shown in Figure 8b, in the case of the green-colored data, additional small-multiple peaks were observed, indicating that their phase purity is worst among the samples. Only the self-doped CsPbI_3_ ($E_{g}$ = 2.59 eV) with the additive ODT displayed a significant blue shift in UV-vis spectra in Figure 5b,d, indicating that the useful black γ-phase might have been minimized when ODT was employed as an organic additive for the self-doped CsPbI_3_.

**Figure 9:**
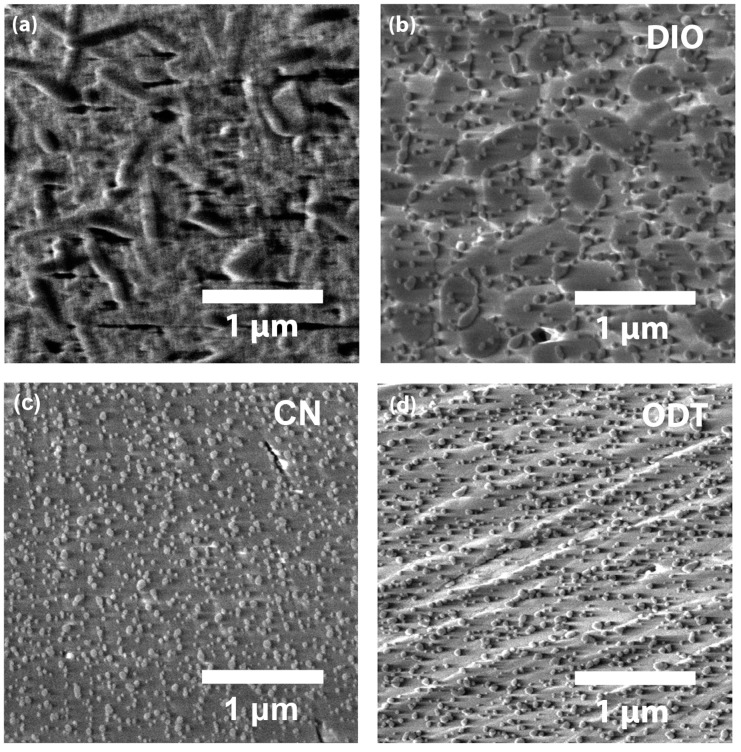
SEM images: (**a**) self-doped CsPbI_3_, (**b**) self-doped CsPbI_3_ with DIO, (**c**) self-doped CsPbI_3_ with CN, and (**d**) self-doped CsPbI_3_ with ODT.

Figure 9 shows a SEM image of the self-doped CsPbI_3_ without/with organic additives DIO, CN, and ODT. The self-doped CsPbI_3_ without additive displays the rod-like textural morphology (Figure 9a) whereas the self-doped CsPbI_3_ with additives shows some common nanoscale spots on the surface of films (Figure 9b–d), which is in line with Kim et al.’s report [96]. However, depending on the additive species, the morphology is somewhat different. The self-doped CsPbI_3_ with DIO shows multiple domains whereas the sample with CN exhibits a relatively flat image. Interestingly, the self-doped CsPbI_3_ with ODT displays some abnormal texture in the diagonal direction (flow-like image), indicating that the film is not uniform because of the ODT’s special character (e.g., probably the ionizability of ODT; in this case, there might be coulombic interactions and chemical reactions between thiol with haloplumbate in the perovskite precursor solution state). AFM tapping mode images can be found in Figure S4, in which the self-doped CsPbI_3_ with ODT shows a rod-like shape instead of granular spots, indicating that the sample could be nonuniform (from different degrees of phase transformation) when processed in air.

**Figure 10:**
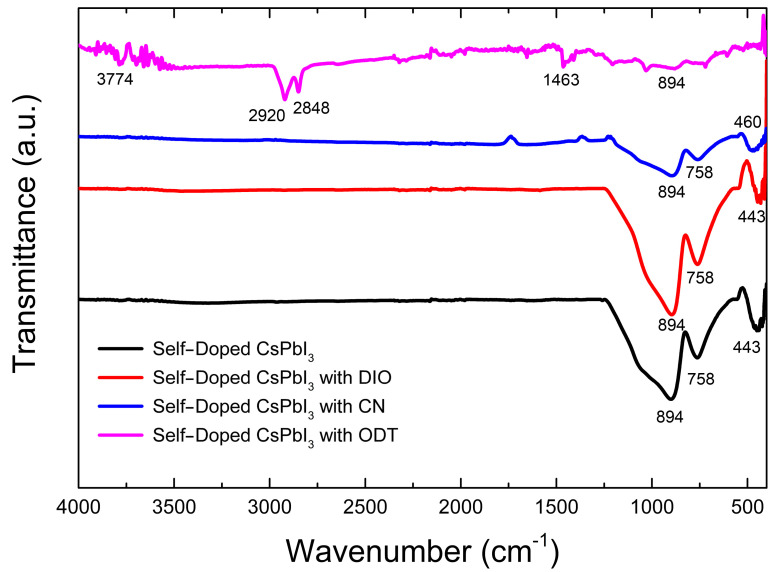
FTIR spectra of self-doped CsPbI_3_ without and with organic additives, DIO, CN, and ODT.

Finally, we investigated whether or not the organic additive stays with the self-doped CsPbI_3_ based on FT-IR spectroscopy. Accordingly, we obtained Figure 10, explaining the functional group in the sample compounds. Largely speaking, the self-doped CsPbI_3_ without/with additive (DIO or CN) showed characteristic FT-IR spectra with three main peaks at 894 cm^−1^, 758 cm^−1^, and ~443 cm^−1^ [97], indicating that DIO and CN molecules, like other solvents such as DMF/DMSO, did not stay in the perovskite film after thermal annealing at 120 °C for 10 min (within the detection limit of IR instrument). However, in the case of the ODT-added sample, the film displays several additional peaks at 3774 cm^−1^, 2920–2848 cm^−1^, and 1483 cm^−1^, originating from O–H vibration (from absorbed water) and/or C–C stretching vibration, C–H stretching, and C–H scissoring, respectively [98,99,100,101]. The presence of C–H vibration from ODT’s alkyl moiety indicates that ODT could be ionized (i.e., R–SH→R–S^−^ + H^+^) and reacted with the perovskite precursor (haloplumbate). Note that alkylthiol (e.g., ODT) has been used to form a self-assembled monolayer on the surface of metal nanoparticles through the reaction between thiolate anion (RS^−^) and metal (e.g., Au) [102], suggesting the binding reaction between thiolate anion and haloplumbate (specifically, soft lead element).

## 4. Conclusions

We demonstrated that self-doped CsPbI_3_ with CsI:PbI_2_ = 2:1 could be useful for retaining a black γ-phase mixture by escaping a pure yellow non-perovskite δ-phase under ambient conditions. In addition, when DIO and CN were employed as an organic additive, the crystallization process was partially modified, leading to the energy bandgap of 1.78 eV (DIO) and 1.76 eV (CN) instead of 1.83 eV (without additive). However, in the case of the CsPbI_3_ with ODT, the bandgap becomes wider, e.g., ~2.59 eV, which might originate from the ionizability of ODT affecting the crystallization process. Interestingly, the PL spectra shows two emission peaks and the PL decay curves displayed two steps, suggesting a compound mixture, about which HR-TEM showed the embedded nanodomains in a crystalline matrix. In the case of additive-solvent’s phase behavior, the predicted miscibility is CN:DMF (or DMSO) > ODT:DMF (or DMSO) > DIO:DMF (or DMSO) based on the Flory–Huggins theory. Specifically, the additive is less miscible with DMSO, indicating that the presence of additive (DIO, CN, and ODT) makes the solvent medium poorer than that without additive. Future work may include the tunability of the phase purity for self-doped CsPbI_3_ perovskite under ambient conditions for perovskite solar cell applications. Finally, considering the interchangeability between two orthorhombic phases (γ-phase and δ-phase) and between two crystalline compounds (Cs_4_PbI_6_ and CsPbI_3_), our results provide insight into the stability of γ-phase CsPbI_3_ perovskite thin film. In other words, the meta-stable γ-phase thin film processed under a controlled environment (e.g., N_2_-filled glove box) undergoes a phase transformation and thereby the phase purity decreases with time.

## Figures and Tables

**Table 1:** Group contribution to $E_{coh}$ (cohesive energy) for estimating the solubility parameter (*δ*) [75,76]. Here, $E_{coh}^{group}$ and $E_{coh}$ are cohesive energy per group and per entire molecule, respectively. $MW_{2}$ is molecular weight, $\rho_{2}$ is density, $V_{2}$ is molar volume of organic additives, b.p. is boiling point, and $\delta_{2}\;\left(\mathrm{or}\;\delta_{2}^{\prime }\right)$ is solubility parameter, respectively. Here, the subscript-2 denotes additive molecule whereas subscript-1 is used for solvent in Table 2.

Additive	Group	$E_{coh}^{group}$ (J/mol)	Group Number	$E_{coh}$ (J/mol)	$MW_{2}$ (g/mol)	$\rho_{2}$ (g/cm^3^)	$V_{2}$ (cm^3^/mol)	b.p. (°C)	$\delta_{2}^{\prime }$ (MPa)^1/2^	$\delta_{2}$ (cal/cm^3^)^1/2^
DIO	-CH_2_-	4190	8	71,620	366.02	0.818	447.5	167–169	19.0	9.3
	-I	19,050	2							
CN	-Cl	12,990	1	58,056	162.62	1.194	136.2	111–113	20.7	10.1
	-CH=CH-	10,200	3							
	>C=C(H)-	4860	1							
	>C=C<	9606 ^a^	1							
ODT	-CH_2_-	4190	8	77,050	178.36	0.970	183.9	269–270	18.8	9.2
	-S-	8800	2							
	-H	12,965 ^a^	2							

^a^ Estimated from the solubility parameter data in the literature [77,78].

**Table 2:** Properties of solvents and antisolvent. $MW_{1}$ is molecular weight, $\rho_{1}$ is density, ${\hat{V}}_{1}$ is molar volume of solvent, b.p. is boiling point, $\delta_{1}\;\left(\mathrm{or}\;\delta_{1}^{\prime }\right)$ is solubility parameter [75], and *D_N_* is Gutmann’s donor number [73], respectively. Here, the subscript 1 denotes solvent molecule.

Solvent	$MW_{1}$ (g/mol)	$\rho_{1}$ (g/cm^3^)	${\hat{V}}_{1}$ (cm^3^/mol)	b.p. (°C)	$\delta_{1}^{\prime }$ (MPa)^1/2^	$\delta_{1}$ (cal/cm^3^)^1/2^	*D_N_* (kcal/mol)
DMF	70.09	0.948	73.9	153	24.8	12.1	26.6
DMSO	78.13	1.100	71.0	189	29.7	14.5	29.8
CB	112.56	1.110	101.4	132	19.5	9.5	3.3

**Table 3:** Flory–Huggins interaction parameter ($\chi_{12}$) and molar volume ratio ($r_{2}=V_{2}/{\hat{V}}_{1}$) when $r_{1}=1$ for the solvent such as DMF or DMSO.

System	DIO:DMF	CN:DMF	ODT:DMF	DIO:DMSO	CN:DMSO	ODT:DMSO
$\chi_{12}$	291.6/*T*	148.8/*T*	312.8/*T*	966.2/*T*	691.8/*T*	1003.7/*T*
$r_{2}*$	6.1	1.8	2.5	6.3	1.9	2.5

* Note that $r_{2}\leq 10$ indicates that the component 2 can be treated as a model oligomer.

## Data Availability

The datasets used and/or analyzed during the current study are available from the corresponding author upon reasonable request.

## Supplementary Materials

The following are available online at: The following supporting information can be downloaded at: https://www.mdpi.com/article/10.3390/mi14081601/s1; Figure S1: Flory–Huggins interaction parameter at 298 K as a function of organic additive species. Figure S2: Crystal structure of trigonal Cs4PbI6 with space group R-3c. Figure S3: High-resolution TEM images of cesium lead iodide thin films: (a) without organic additive and (b) with organic additive, ODT. Figure S4: AFM tapping-mode height image. (a) Self-doped CsPbI_3_ without any additive, (b) self-doped CsPbI_3_ with DIO, (c) self-doped CsPbI_3_ with CN, and (d) self-doped CsPbI_3_ with ODT. Table S1: Flory–Huggins χ12 interaction parameter at 298 K as a function of organic additive species.
