# Supplementary material for: Phase Behavior and Role of Organic Additives for Self-Doped CsPbI3 Perovskite Semiconductor Thin Films
Source: Micromachines (Basel). 2023 Aug 14;14(8):1601. doi: 10.3390/mi14081601 (PMC10456489; doi:10.3390/mi14081601)
Supplement: Supplementary file 1 [file micromachines-14-01601-s001.zip › micromachines-2538828-supplementary.pdf]

micromachines

Supplementary Materials

Phase Behavior and Role of Organic Additives for Self-Doped $CsPbI_3$ Perovskite Semiconductor Thin Films

Tamiru Kebede[1,2], Mulualem Abebe [1], Dhakshnamoorthy Mani [1], Jibin Keloth Paduvilan [3], Lishin Thottathi [4], Aparna Thankappan [5], Sabu Thomas [6], Irfan Anjum Badruddin [7], Sarfaraz Kamangar [7], Abdul Saddique Shaik [7], Fekadu Gochole Aga [8,9] and Jung Yong Kim [8,9,*]

1 Faculty of Materials Science and Engineering, Jimma Institute of Technology, Jimma University, Jimma P.O. Box 378, Ethiopia; tamiruks@gmail.com (T.K.); mulualem.mekonnen@ju.edu.et (M.A.); dhakshnamoor-thy.mani@ju.edu.et (D.M.)
2 Department of Physics, College of Natural and Computational Science, Bonga University, Bonga P.O. Box 334, Ethiopia
3 School of Chemical Sciences, Mahatma Gandhi University, Kottayam 686560, India; jibinkp999@gmail.com
4 Department of Physics and Mathematics, Università Cattolica del Sacro Cuore, Via della Garzetta, 48, 25133 Brescia BS, Italy; lishint123@gmail.com
5 Department of Physics, Baselius College, Kottayam 686001, India; aparnathankappan@baselius.ac.in
6 School of Energy Materials, Mahatma Gandhi University, Kottayam 686560, India; sabuthomas@mgu.ac.in
7 Mechanical Engineering Department, College of Engineering, King Khalid University, Abha 61421, Saudi Arabia; sarfaraz.kamangar@gmail.com (S.K.); abdul.siddique1976@gmail.com (A.S.S.); magami.irfan@gmail.com (I.A.B.)
8 Department of Materials Science and Engineering, Adama Science and Technology University, Adama P.O. Box 1888, Ethiopia; fekadu.gochole@astu.edu.et
9 Center of Advanced Materials Science and Engineering, Adama Science and Technology University, Adama P.O. Box 1888, Ethiopia

* Correspondence: jungyong.kim@astu.edu.et

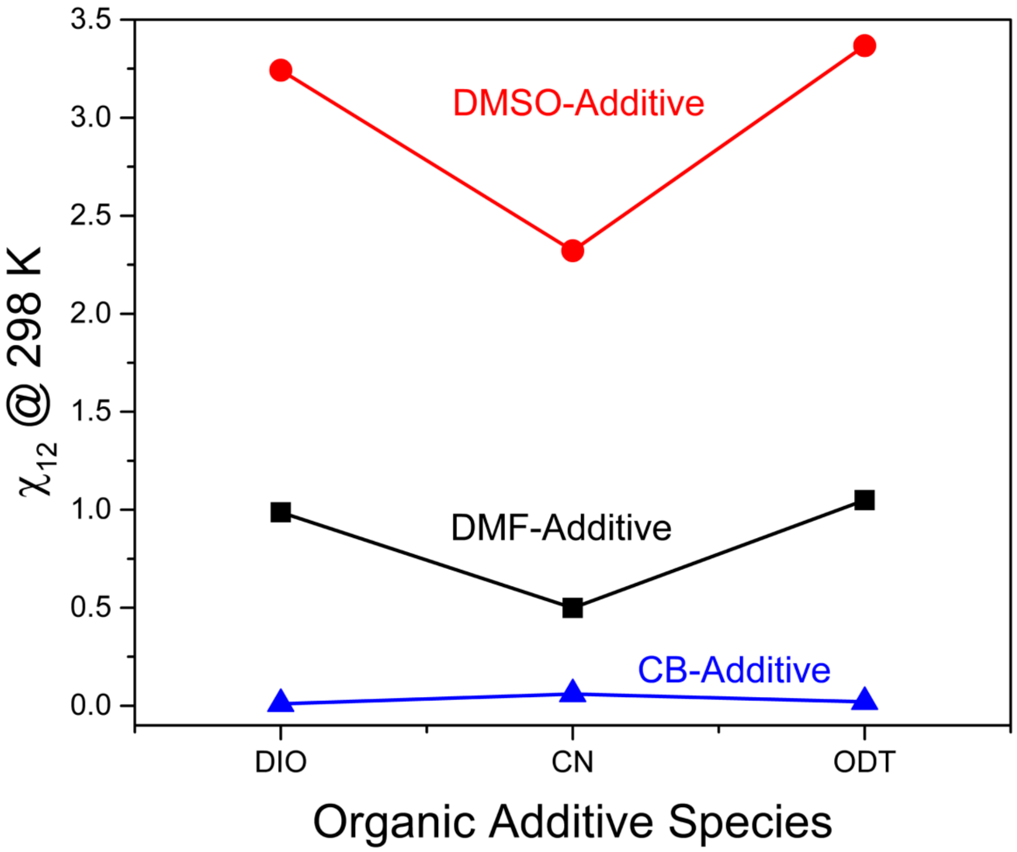

Figure S1. Flory-Huggins interaction parameter at 298 K as a function of organic additive species.

Table S1. Flory-Huggins χ_{12} interaction parameter at 298 K as a function of organic additive species.

	Binary additive-solvent system								
	DMF			DMSO			CB*		
	DIO	CN	ODT	DIO	CN	ODT	DIO	CN	ODT
χ_{12}	0.987	0.499	1.049	3.242	2.321	3.368	0.01	0.06	0.02

* Chlorobenzene (CB) is used as an antisolvent in this study.

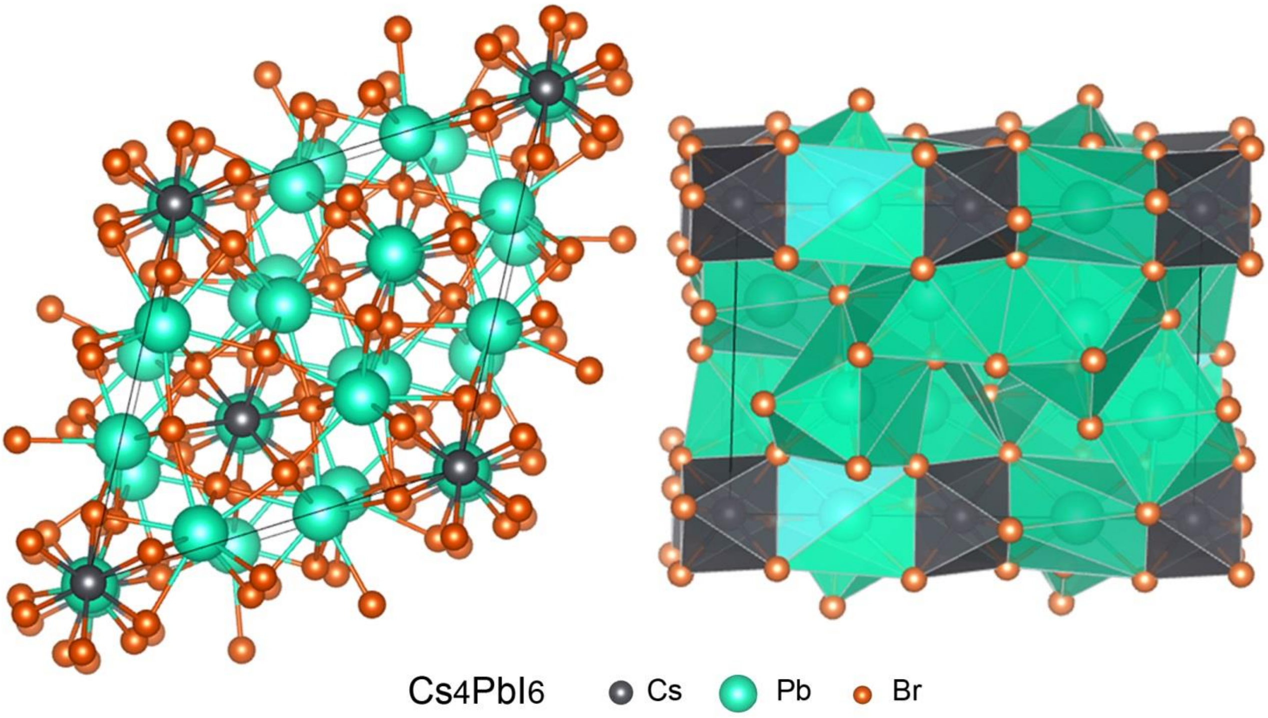

Figure S2. Crystal structure of trigonal Cs_4PbI_6 with space group R-3c.

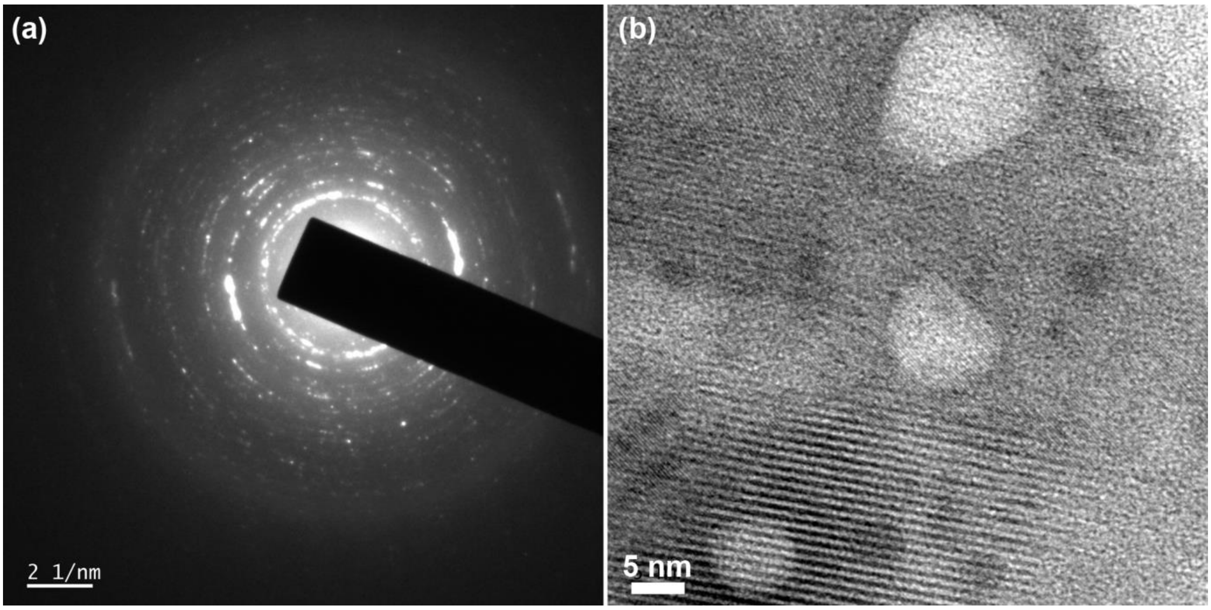

Figure S3. (a) Selected area diffraction pattern and (b) high-resolution TEM images of self-doped $CsPbI_3$ with the organic additive ODT.

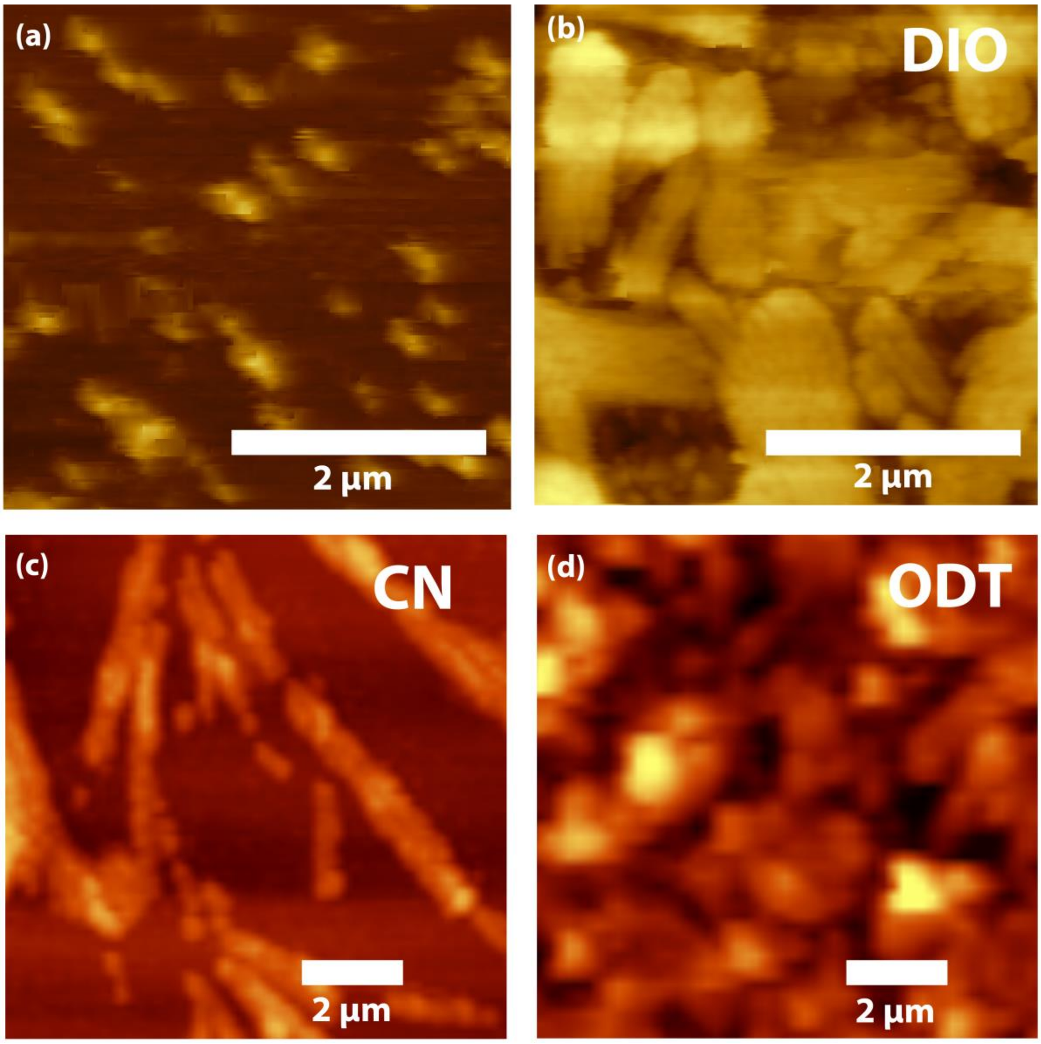

Figure S4. AFM tapping-mode height image. (a) Self-doped $CsPbI_3$ without any additive, (b) self-doped $CsPbI_3$ with DIO, (c) self-doped $CsPbI_3$ with CN, and (d) self-doped $CsPbI_3$ with ODT.
